# Plantecophys - An R Package for Analysing and Modelling Leaf Gas Exchange Data

**DOI:** 10.1371/journal.pone.0143346

**Published:** 2015-11-18

**Authors:** Remko A. Duursma

**Affiliations:** Hawkesbury Institute for the Environment, Western Sydney University, Penrith, NSW, Australia; Wageningen University, NETHERLANDS

## Abstract

Here I present the R package 'plantecophys', a toolkit to analyse and model leaf gas exchange data. Measurements of leaf photosynthesis and transpiration are routinely collected with portable gas exchange instruments, and analysed with a few key models. These models include the Farquhar-von Caemmerer-Berry (FvCB) model of leaf photosynthesis, the Ball-Berry models of stomatal conductance, and the coupled leaf gas exchange model which combines the supply and demand functions for CO_2_ in the leaf. The 'plantecophys' R package includes functions for fitting these models to measurements, as well as simulating from the fitted models to aid in interpreting experimental data. Here I describe the functionality and implementation of the new package, and give some examples of its use. I briefly describe functions for fitting the FvCB model of photosynthesis to measurements of photosynthesis-CO_2_ response curves ('A-C_i_ curves'), fitting Ball-Berry type models, modelling C3 photosynthesis with the coupled photosynthesis-stomatal conductance model, modelling C4 photosynthesis, numerical solution of optimal stomatal behaviour, and energy balance calculations using the Penman-Monteith equation. This open-source package makes technically challenging calculations easily accessible for many users and is freely available on CRAN.

## Introduction

Since the advent of portable gas exchange instruments [1,2], a wealth of data on leaf gas exchange of CO_2_ and H_2_O has been collected [3]. These data play a central role in physiological plant ecology [4], to better understand and quantify inter-specific differences in photosynthesis and transpiration, and to quantify and model the rapid response to changes in environmental drivers such as light, humidity and temperature. Not only do leaf gas exchange data allow detailed studies of the underlying plant physiology, they are also used to parameterize an important component of process-based models of vegetation function used to predict global water and carbon cycling [5,6].

The photosynthesis model of Farquhar, von Caemmerer and Berry [7] (the 'FvCB model') is widely used in interpreting and modelling leaf gas exchange, by providing comparable metrics of the photosynthetic capacity, and predicting the response of photosynthesis to changes in the CO_2_ concentration inside the leaf air space (C_i_). This widely cited model is embedded in many process-based models of vegetation function [5,8]. The key prediction of the model is the response of photosynthesis to [CO_2_] inside the leaf (either chloroplastic [CO_2_], C_c_, or intercellular [CO_2_], C_i_). It can also account for changes in leaf temperature if the various temperature sensitivities are parameterized [9–11]. To employ the model, it is generally fit to observations of net photosynthesis along a range of [CO_2_] concentrations, yielding well-known measures of photosynthetic capacity (V_cmax_ and J_max_, and optionally R_d_) [12].

**Fig 1:**
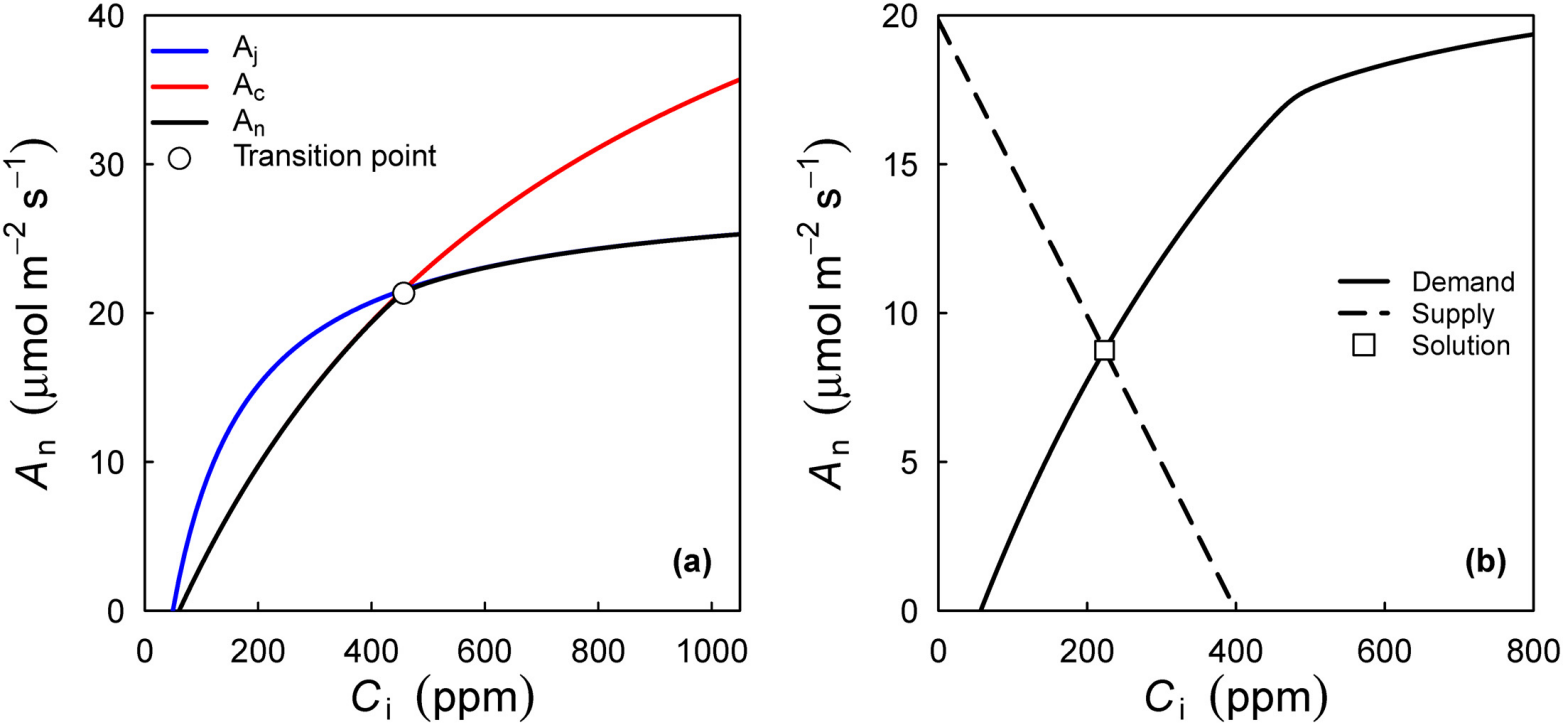
Supply and demand functions for leaf photosynthesis. (a) Leaf photosynthesis—CO_2_ response curve as modelled with the FvCB model. (b) The intersection of the supply and demand curves of photosynthesis. The Photosyn function solves for C_i_ if g_s_, V_cmax_, J_max_ and R_d_ (and other parameters to the FvCB model) are known.

I do not repeat a detailed description of the FvCB model here, as it has been described many times [10,11]. But generally it is of the form,

$${\text{A}}_{\text{n}}=\text{min}\left({\text{A}}_{\text{c}},{\text{A}}_{\text{j}}\right)-{\text{R}}_{\text{d}}$$ (1)

where A_n_ is the net rate of CO_2_ assimulation, A_c_ is the gross photosynthesis rate when Rubisco activity is limiting, A_j_ when RuBP-regeneration is limiting, and R_d_ the rate of dark respiration (see Fig 1A). A_c_ and A_j_ are non-linear functions of the chloroplastic CO_2_ concentration (C_c_), both of the form k_1_ (C_c_Γ*)/(k_2_+C_c_), where Γ* is the CO_2_ compensation point without R_d_, and k_1_ and k_2_ are different parameter combinations for A_c_ and A_j_. The details of these functions and the temperature dependence of the various parameters are described elsewhere [11].

In the practical application of the FvCB model, when leaf gas exchange is measured with a portable gas exchange instrument, estimates of C_c_ are difficult to obtain because they require an estimate of the mesophyll conductance (g_m_). In this case, it is customary to us the intercellular [CO_2_] concentration (C_i_) as the driver of photosynthesis. This approach is useful because C_i_ can be estimated from concurrent measurements of CO_2_ and H_2_O flux [13]. In the remainder of this article I will use C_c_ as the driver of photosynthesis, but point out that this can be replaced by C_i_ if the user does not have an estimate of g_m_. When g_m_ is known, C_c_ is calculated from C_i_ with Eq 2.

$${\text{C}}_{\text{c}}={\text{C}}_{\text{i}}-{\text{A}}_{\text{n}}/{\text{g}}_{\text{m}}$$ (2)

where g_m_ is the mesophyll conductance (mol m^-2^ s^-1^). Although this method assumes that g_m_ is constant for a given leaf, it is well known that g_m_ responds dynamically to fluctuations in environmental drivers [14] although some of the variation in g_m_ may due to artefacts related to (photo-)respiratory effects on measured g_m_ with standard methods [15]. Because no model has been developed to date that adequately captures the variation in g_m_, it is a constant parameter in the implementation presented here. However, it can still be used to study the effects of non-constant g_m_ on rates of photosynthesis and its response to environmental drivers, as the parameter can be varied in model simulations.

Fitting the FvCB model to data requires some finesse because net photosynthesis is modelled as a minimum function of two non-linear equations that is sometimes difficult to fit. Moreover, sample sizes collected are often small due to time constraints in the field. A widely used published method requires the user to specify the transition of V_cmax_ to J_max_ limitation [16], a process that is both arbitrary and prevents batch analysis. Another method [17] requires online submission of data and fits the model without much control or knowledge of the fitting process (following [18]), and does not report standard errors of the estimated parameters. Undoubtedly many more implementations of the fitting process have been developed over the years, but few of these are made publicly available (but see available online tools [19,20]). What is missing is an open-source tool that can be used for reproducible and transparent analysis of A-C_i_ curves.

Through Eq 1, we have a dependency of photosynthesis on the availability of the substrate, C_c_. To estimate C_c_ itself, we need C_i_, which can be estimated when with stomatal conductance to CO_2_. From Fick's law, we can relate A_n_ to g_s_ and C_i_ as,

$${\text{A}}_{\text{n}}=\frac{{\text{g}}_{\text{s}}}{1.6}\left({\text{C}}_{\text{a}}-{\text{C}}_{\text{i}}\right)$$ (3)

where g_s_ is the conductance to H_2_O (the factor 1.6 converts to conductance to CO_2_). We now have two equations for A_n_: the 'demand function' (Eq 1), and the 'supply function' (Eq 3). At steady state these two equations should be equal, which can be graphically shown as in Fig 1B (cf. [21]).

Because g_s_ itself responds to environmental drivers, another expression is needed to end up with a fully coupled model of leaf gas exchange. The most widely-used, though empirical, g_s_ model is the Ball-Berry [22] class of models. This model posits an entirely empirical equation that describes the response of g_s_ to air humidity, CO_2_ and A_n_. This way, effects of leaf temperature and PPFD—both of which are known to affect g_s_—are modelled through the dependency of A_n_ on these drivers. A general form of the Ball-Berry model is,

$${\text{g}}_{\text{s}}={\text{g}}_{0}+{\text{g}}_{1}\frac{{\text{A}}_{\text{n}}}{{\text{C}}_{\text{a}}}\text{f}\left(\text{D}\right)$$ (4)

where D is the vapour pressure deficit (kPa), g_0_ and g_1_ are empirical parameters, and f(D) can be one of many functions that describe the response to the vapour pressure deficit (D, [23,24]) or relative humidity [22]. An alternative approach to modelling g_s_ is through the hypothesis that stomata act optimally in the sense that they maximize photosynthesis while minimizing water loss. This hypothesis was first developed by Cowan and Farquhar [25] and has seen many applications. Medlyn et al. [24] showed that the optimality hypothesis, when coupled to the FvCB model, leads to an expression analogous to the Ball-Berry type models (Eq 4), but with a different D response function (f(D) in Eq 3) compared to the original Ball-Berry model.

Finally we can combine the biochemical demand function of photosynthesis (Eq 1) with the supply function (Eq 2) and an expression for the dependency of g_s_ on environmental drivers (Eq 3). This 'coupled' leaf gas exchange model [23,26,27] is implemented in many process-based ecosystem and global land surface models [5,8,28,29]. This model allows prediction of A_n_, g_s_ and leaf transpiration rate in response to all major environmental drivers (except soil water limitation), and incorporates key leaf traits (g_1_, V_cmax_, J_max_, R_d_, and their temperature dependencies).

Despite the widespread use of the FvCB model and the coupled leaf gas exchange model, tools to analyse data and perform simulations are scattered and subject to little standardization. Fitting the FvCB model to CO_2_ response curves is a standard procedure but different methods can yield different parameter values, making comparisons difficult. The coupled leaf gas exchange model is not straightforward to implement, and I do not know of any standalone open-source implementations. I here describe the plantecophys package, implemented in the R language [30]. The code is freely available (without restrictions), and managed with a version control system. The package is the result of our work on leaf and canopy modelling of photosynthesis and stomatal conductance [24,31–36], with many additions based on user requests.

## Design and Implementation

### The main functions

The main tools included in the plantecophys package are to a) fit A-C_i_ curves to estimate V_cmax_, J_max_ and R_d_, b) fit Ball-Berry type models, c) simulate from the coupled leaf gas exchange model and d) calculate the optimal stomatal conductance. The key functions in the package are summarized in Table 1.

**Table 1:** Main functions in the plantecophys package.

Function	Description
fitaci	Fit, summarize, plot and simulate photosynthesis-[CO^2^] response curves (A-Ci curves)
fitBB	Fit Ball-Berry type models of stomatal conductance
FARAO	Estimate optimal stomatal conductance with a numerical implementation of the Cowan-Farquhar hypothesis
Photosyn	Simulate C3 photosynthesis and transpiration with the coupled leaf gas exchange model. Also simulates the FvCB model when either C^i^ or g^s^ is given as input.
PhotosynEB	Estimate leaf temperature from energy balance, when a significant leaf boundary layer is present
AciC4	Simulates the dependence of C4 photosynthesis on the intercellular CO^2^ concentration
RHtoVPD etc.	Convert between commonly used units (relative humidity, vapour pressure deficit, dewpoint temperature)

### Language

The 'plantecophys' package is implemented in R, has no dependencies on other packages, and does not require compilation (i.e. it is written in native R only). As such it builds easily, and is highly portable. The source code is maintained with git version control, and is hosted in an online repository (http://www.bitbucket.org/remkoduursma/plantecophys), from which a development version of the package can easily be installed. The repository includes an issue tracker, where users can suggest changes or report bugs. This paper describes version 0.6.6 (git SHA b9a18c9).

All code used in this article (including the code to generate the article written in markdown, all figures and full example code), can be downloaded from Ref. [37]. The repository also includes code to demonstrates how to extract additional statistics from fitted A-C_i_ curves.

## Results and Discussion

### Fitting A-C_i_ curves

The fitaci function fits the FvCB model, yielding estimates of V_cmax_, J_max_ and R_d_ and their standard errors. Instead of fitting the minimum function (Eq 1), fitaci fits the hyperbolic minimum of A_c_ and A_j_, which avoids a discontinuity (Eq 5).

$${\text{A}}_{\text{m}}=\frac{{\text{A}}_{\text{c}}+{\text{A}}_{\text{j}}-\sqrt{{\left({\text{A}}_{\text{c}}+{\text{A}}_{\text{j}}\right)}^{2}-4{\theta \text{A}}_{\text{c}}{\text{A}}_{\text{j}}}}{2\theta }-{\text{R}}_{\text{d}}$$ (5)

where θ is a shape parameter, set to 0.9999, and A_m_ is the hyperbolic minimum of A_c_ and A_j_. The fit of the FvCB model to data is achieved with non-linear least squares, and standard errors of the parameters are estimated with standard methods (nls function in base R, see [38]). The fitaci function includes methods to estimate appropriate starting values from the data, and attempts the fits along a wide range of possible starting values. Optionally, R_d_ can be provided as a known value, otherwise it is estimated from the A-C_i_ curve. The user does not have to provide the transition point (see Fig 1), as this is estimated by fitaci automatically. It is however an option to fix the transition point (via the citransition argument), which may be helpful to check whether the best fit was achieved. Finally, the user can provide an estimate of mesophyll conductance (g_m_) (following [39]), in which case the fitted values of V_cmax_ and J_max_ can be interpreted as chloroplastic rates.

Because the fitting uses non-linear least squares, standard methods can be employed to estimate standard errors (SE), confidence intervals, and correlation of the fitted parameters. The fitaci function returns by default the SE and confidence intervals, and the built-in help page for the fitaci function shows how the nlstools package can be used to provide a detailed overview of the statistics of the non-linear least squares fit.

Required inputs are measurements of A_n_ and C_i_, and optionally leaf temperature (T_leaf_), and photosynthetically active radiation (PAR). Also required are estimates of Michaelis-Menten constants (K_c_, K_o_ or the combination K_m_) and Γ*. In the FvCB model, J_max_, V_cmax_ and leaf respiration (R_d_) (and other parameters like Γ*, K_c_ and K_o_) all depend non-linearly on T_leaf_. The Photosyn function incorporates standard temperature sensitivities for all parameters of the FvCB model (following [11]). Optionally, measured (or otherwise modelled) K_m_ and Γ* can be provided as input.

The function takes a dataframe as input, which includes measurements of A_n_, C_i_ and optionally T_leaf_ and PAR, and is easily used like this:

# Fit FvCB model

f <- fitaci(mydata)

# Print a summary with coefficients and more

f

# Make standard plot

lot(f)

**Fig 2:**
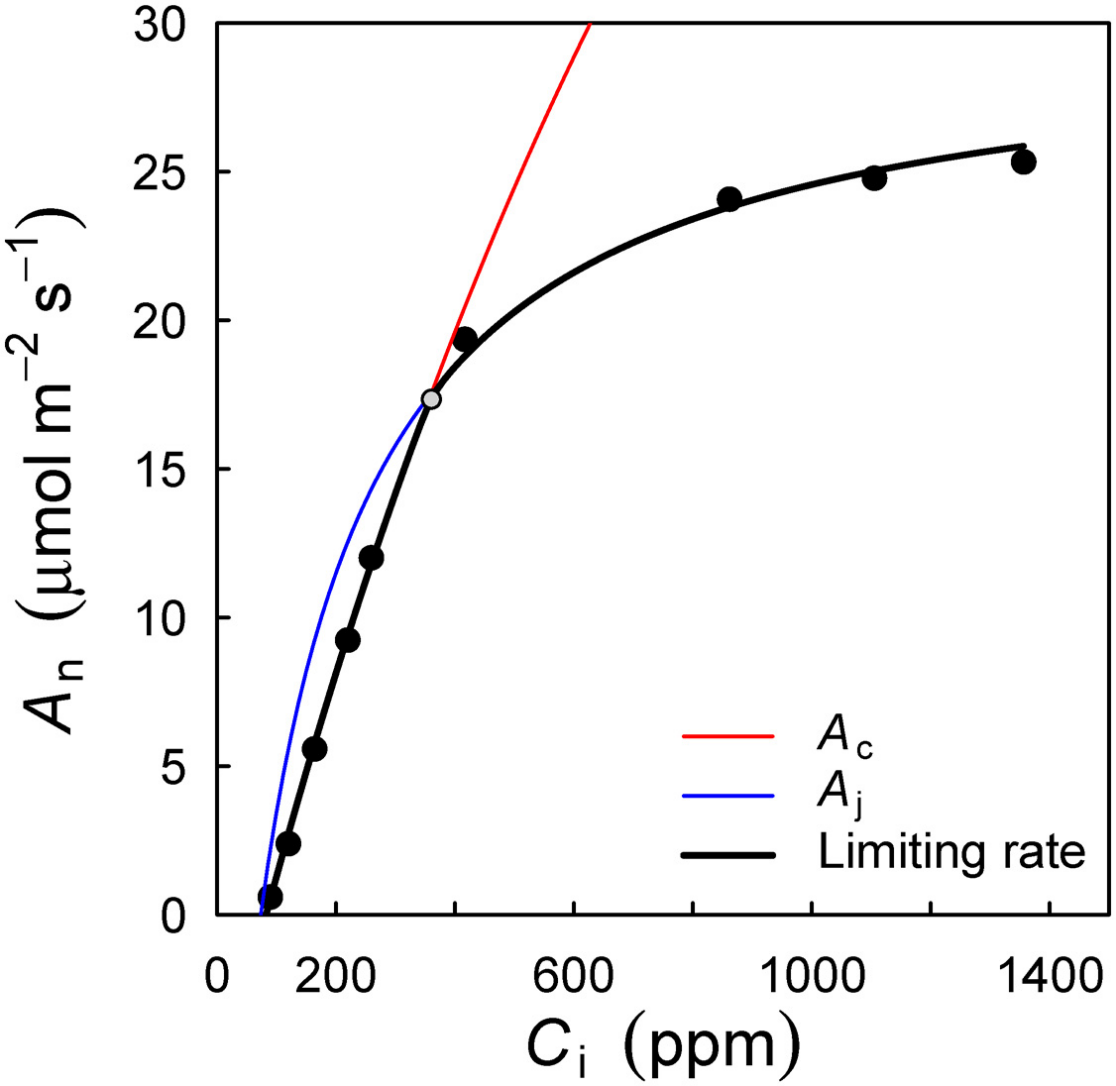
Standard output from the fitaci function. A_n_ is the net photosynthetic rate, C_i_ the intercellular CO_2_ concentration. Symbols are measurements, the black line the fitted FvCB model of photosynthesis. Colored lines indicate the two photosynthesis rates in the FvCB model. In the default mode, the fitaci function estimates V_cmax_, J_max_ and R_d_ from the fitted curve. Optionally, R_d_ is provided as an input, for example when it was measured separately. In this example, V_cmax_ was estimated as 46.8 (SE 1.47), J_max_ was 105.2 (SE 1.36) and R_d_ was 1.3 (SE 0.24). Assumed parameters were K_m_ = 1460 and Γ* = 64.8 (all in units of μmol m^-2^ s^-1^). The R^2^ of a regression of measured vs. fitted was 0.99.

The output of the above example is shown in Fig 2. Additionally, the batch utility fitacis can be used to fit many curves at once, for example one for each species or site in a dataset. I show this functionality in the example application further below.

A C4 model of leaf photosynthesis [40] is also implemented (in AciC4), but at the moment it is only possible to fit the C3 model of leaf photosynthesis to A-C_i_ curves.

### Fitting stomatal conductance models

The straightforward fitBB function provides an interface to non-linear or linear regression to fit one of three stomatal conductance models [22–24]. This yields estimates of g_1_ and (optionally) g_0_, which are necessary inputs to the coupled leaf gas exchange model. Note that the user must provide stomatal conductance to H_2_O (not CO_2_) as input to the fitting process, which is the standard output of portable gas exchange instruments. This function is demonstrated in the example application further below.

### Coupled leaf gas exchange model

The intersection of the supply and demand curves of photosynthesis (Fig 1B) gives the steady-state intercellular CO_2_ concentration (C_i_). This is solved by the Photosyn function. This flexible interface can be used to either 1) estimate A_n_ when C_i_ is known (Photosyn(Ci = …); equivalent to Aci(…)), 2) estimate A_n_ when g_s_ is known (Photosyn(GS = …)) (cf. Fig 1B) or c) solve for C_i_ from the coupled leaf gas exchange model (Eqs 1,3 and 4).

**Fig 3:**
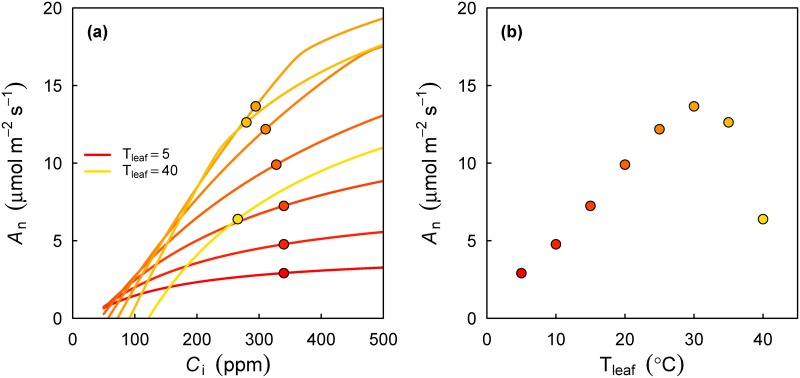
Response of A_n_ and C_i_ to combined changes in T_leaf_ and D. (A) Lines are A-C_i_ curves simulated at a range of values for T_leaf_. Symbols are the solutions of the coupled leaf gas exchange model, while also taking into account the correlation between D and T_leaf_ (based on an empirical relationship [35]: D = 0.000605*T_air_^2.39^). Note that as T_leaf_ and D increase, C_i_ decreases. (B) The corresponding temperature optimum of A_n_. Symbols are the same as in panel (A) but plotted against T_leaf_.

To demonstrate the use of the coupled gas exchange model, I visualize the temperature response of A_n_ when both T_leaf_ and D are varying. In field conditions, D is always strongly positively related to T_leaf_. The consequence is that when studying D or T_leaf_ responses in the field, both drivers have to be accounted for simultaneously [35,41]. Fig 3B shows simulated A-C_i_ curves and the solutions of the coupled leaf gas exchange models at a range of T_leaf_ and corresponding D (calculated following [35]). Both V_cmax_ and J_max_ have a peaked response to T_leaf_, so that at a given C_i_, A_n_ first increases with T_leaf_ and then decreases (lines, Fig 3A). As a result of increasing D, the modelled C_i_ decreases (symbols, Fig 3A, as a consequence of Eq 4). The net result is a peaked response of A_n_ as a function of D (Fig 3B).

The simplified code to produce Fig 3B, using the Photosyn function, is given below. Note that in this example the default values of many parameters (e.g. J_max_, g_1_) are used in the call to Photosyn, but all of these can be set by the user.

# Set range of leaf temperature

tleafs <- seq(5, 40, by = 5)

# Define D as a function of Tleaf

vpdfun <- function(tair)0.000605*tair^2.39

# Simulate.

run1 <- Photosyn(Tleaf = tleafs, VPD = vpdfun(tleafs))

# Plot (produces Fig 3b minus the special formatting)

with(run1, plot(Tleaf, ALEAF))

The Photosyn function assumes that the boundary layer conductance (g_bl_) is high compared to g_s_, so that T_leaf_ is close to T_air_. As an alternative, the PhotosynEB function calculates T_leaf_ from the leaf energy balance. Transpiration is calculated with the Penman-Monteith equation [42], which accounts for boundary layer effects. The details of PhotosynEB are not described here (see the built in help file for more information), because it is very similar to other implementations [28,43].

### Numerical solution of optimal stomatal conductance

The FARAO function (FARquhar And Optimality) calculates optimal stomatal conductance based on the Cowan-Farquhar [25] hypothesis that stomata respond to environmental drivers in order to maximize photosynthesis while minimizing water loss. This implementation was used by Medlyn et al. [24] to compare a simplified model of optimal stomatal conductance to the full numerical solution.

**Fig 4:**
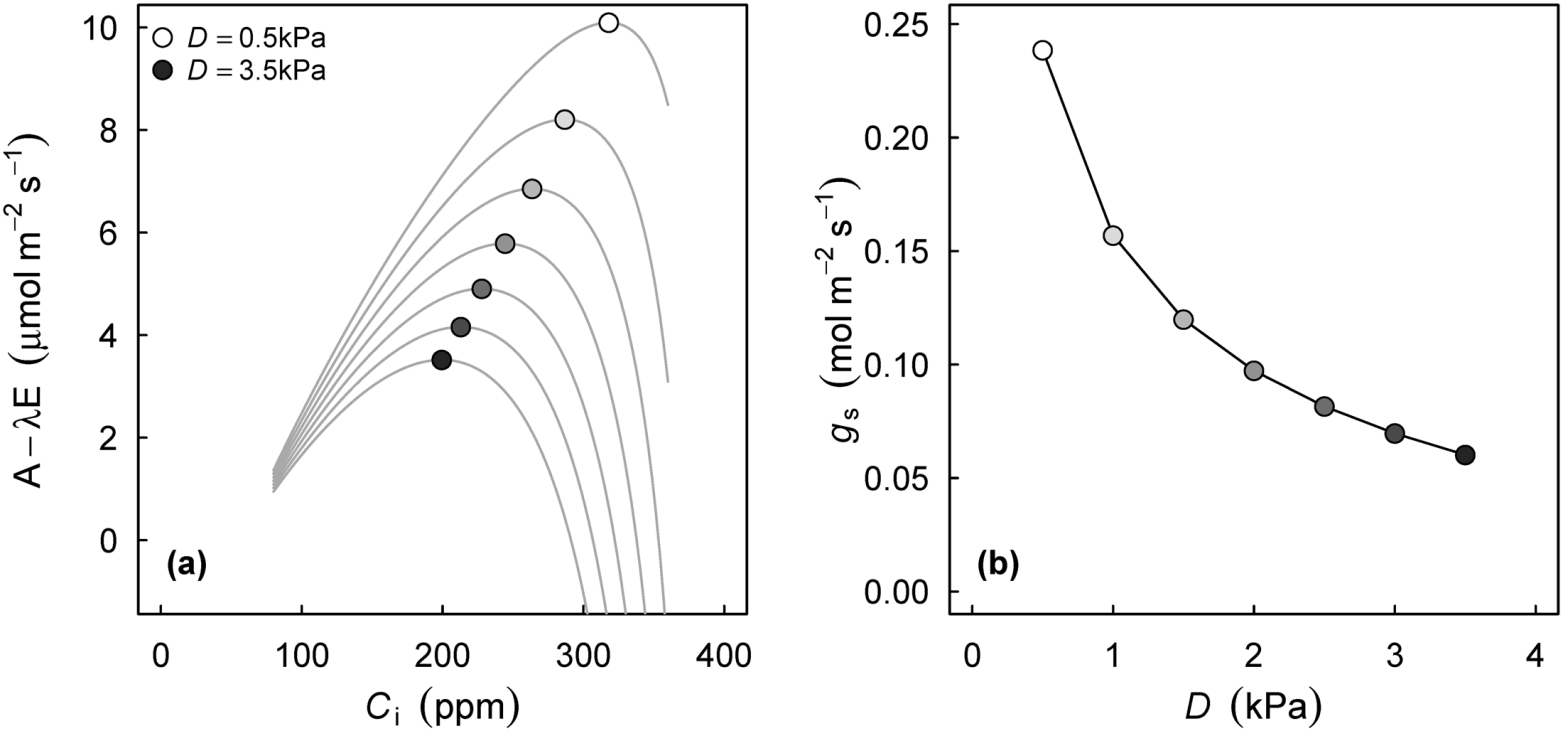
Visualization of the optimal model of stomatal conductance. Provided we have an estimate of the 'cost of water' (λ, mol C mol H_2_O^-1^), stomata act to maximize photosynthesis minus transpiration. In (A), individual curves at a range of values for the vapour pressure deficit (D) are plots of A−λE as a function of C_i_, demonstrating that an optimum C_i_ exists. The FARAO function finds this optimum numerically and calculates corresponding A_n_ and g_s_. The corresponding response of g_s_ to D is shown in panel (B).

To find optimal stomatal conductance, FARAO finds the C_i_ for which the quantity A_n_−λE is maximal, where E is the leaf transpiration rate and λ is the marginal cost of water (an empirical parameter related to g_1_, see [24,25]). A_n_ is calculated directly as a function of C_i_ via the FvCB model (Eq 1), g_s_ is calculated by rearranging Eq 3, and E is calculated assuming perfect coupling (thus E = g_s_D/P_a_, where P_a_ is atmospheric pressure). This numerical routine does not need specification of an f(D) function as in Eq 4, instead, this function is an emergent property. In Fig 4A, I have calculated A_n_−λE across a range of C_i_ values, and for different values of D. The FARAO function calculates the optima of these curves, which can for example be used to study the response of stomatal conductance to D (Fig 4B).

Optionally, the FARAO function accounts for the presence of a leaf boundary layer (when energybalance = TRUE). In that case it uses PhotosynEB (see description above) to calculate A_n_ and E, and solves for T_leaf_. A very similar method was employed by Buckley et al. [43], who demonstrated that when a boundary layer is present, frequently an optimal g_s_ cannot be found.

### An example application

**Fig 5:**
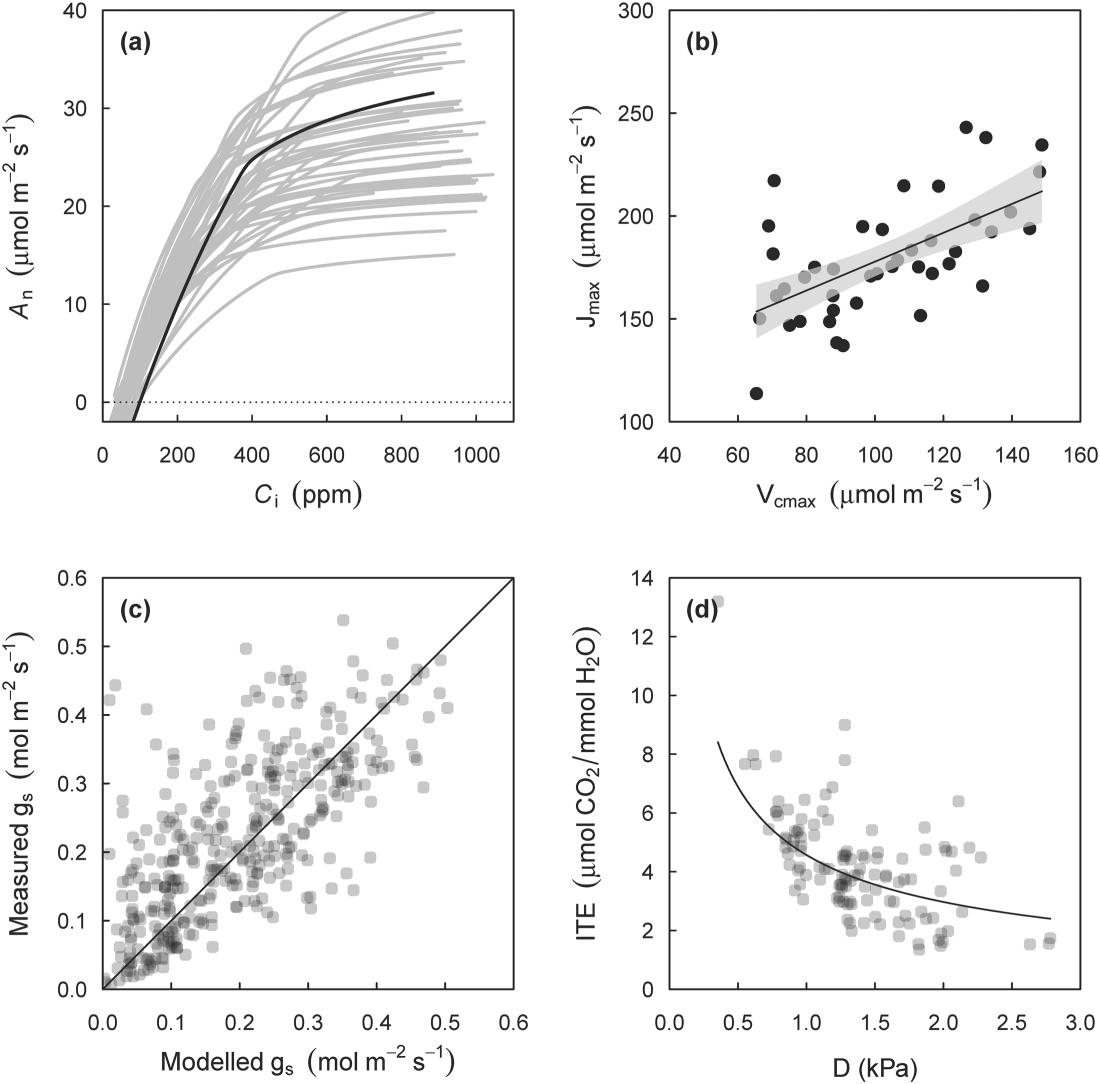
Example application of the plantecophys package to A-C_i_ curves and spot gas exchange measurements on *Eucalyptus delegatensis*. (A) Fitted A-C_i_ curves with one curve highlighted (B) Estimates of J_max_ plotted against V_cmax_, obtained from the fitted curves in panel (A). Solid line is a regression line (J_max_ = 107.71 + 0.7 V_cmax_, R^2^ = 0.36) with a 95% confidence interval for the mean. (C) Modelled (with the model of Medlyn et al. 2011) versus measured g_s_ (p < 0.0001, R^2^ = 0.69). Measurements included a wide range of environmental conditions (PAR, T_leaf_, D). In this example, only g_1_ was fit (estimate = 3.31, 95% CI = 3.15–3.47). (D) The predicted response of ITE (A_n_ / E) as a function of D from the fitted model in panel (C) (solid line), and the measurements from panel (C) when PAR > 1000.

To demonstrate a practical application of the key functions in the package, I use field-collected data from Medlyn et al. [44,45] on *Eucalyptus delegatensis*. Both A-C_i_ curves and 'spot gas exchange' data (i.e. leaf gas exchange measurements at prevailing environmental conditions) were collected. Using the fitacis function, it is straightforward to fit all 43 curves to the A-C_i_ data, and make standard plots of the fitted curves (shown in Fig 5A). The fitted coefficients can be extracted using the coef function, and used to plot a comparison of fitted V_cmax_ and J_max_ values, which show the typical correlation between the two (Fig 5B).

Next, I fit Eq 4 to the spot gas exchange data, yielding an estimate of g_1_ (Fig 5C). In this example, I used the model of Medlyn et al. [24], which is given by Eq 6 (in this example, I assumed g_0_ = 0)

$${\text{g}}_{\text{s}}={\text{g}}_{0}+1.6(1+\frac{{\text{g}}_{1}}{\sqrt{(}\text{D})})\frac{{\text{A}}_{\text{n}}}{{\text{C}}_{\text{a}}}$$ (6)

In Fig 5C, modelled g_s_ is compared to measurements. To compare the model prediction of instantaneous transpiration efficiency (A_n_/E) to measurements along the variation in D (Fig 5D), Eq 6 can be rearranged to give (cf. [34], where it is assumed that g_0_ = 0)

$${\text{A}}_{\text{n}}/\text{E}=\frac{{\text{C}}_{\text{a}}{\text{P}}_{\text{a}}}{1.6\left({\text{g}}_{1}{\text{D}}_{\text{s}}^{\text{k}}+{\text{D}}_{\text{s}}\right)}$$ (7)

Because fitBB can fit a number of Ball-Berry type variants, the various models can be easily compared in terms of goodness of fit. This simple example application is available in the published repository (see Methods), and simplified code for this example (panels a-c only), omitting special formatting and a few minor settings, is given below.

# Fit A-Ci curves.

# In this case, each separate curve is indexed by a column named 'Curve',

# and the data were already read into a dataframe (tumh)

acifits <- fitacis(tumh, "Curve")

# Plot all A-Ci curves in one panel, highlight one fitted curve.

plot(acifits, "oneplot", highlight = "25")

# Fit Medlyn et al.'s (2011) version of the Ball-Berry model

# Data are already read into a dataframe (tumspot),

# and have standard names (or they can be set).

gfit <- fitBB(tumspot, gsmodel = "BBOpti")

# Plot measured versus modelled, by predicting from the fitted model.

tumspot$GSpred <- predict(gfit$fit, tumspot)

with(tumspot, plot(GSpred, Cond))

## Conclusions

We need an open source set of tools to analyse leaf gas exchange data, as these data form a cornerstone of plant physiological ecology. At the moment there are no publicly available tools to fit A-C_i_ curves or perform simulations with the coupled leaf gas exchange model that can be used as part of a reproducable workflow. The plantecophys R package is implemented in widely used language for data analysis. The package includes a useful set of tools to perform standard, and more advanced, analyses of leaf gas exchange data. The open source framework combined with version control allows further development of the code.

## Availability and Requirements

**Project name**: plantecophys

**Project Stable Release**: cran.r-project.org/package = plantecophys

**Project Home Page**: http://www.bitbucket.org/remkoduursma/plantecophys

**Project Issue Tracker**: http://www.bitbucket.org/remkoduursma/plantecophys/issues

**Operating System(s)**: Platform Independent

**Programming Language(s)**: R

**Other Requirements**: none
